# Mechanical Properties of Polyethylene/Carbon Nanotube Composites from Coarse-Grained Simulations

**DOI:** 10.3390/nano15030200

**Published:** 2025-01-27

**Authors:** Daniela A. Damasceno, Keat Yung Hue, Caetano R. Miranda, Erich A. Müller

**Affiliations:** 1 Department of Mechatronics and Mechanical Systems Engineering, Polytechnic School, University of São Paulo, Av. Professor Mello Moraes, 2231, São Paulo 05508-030, SP, Brazil; daniela.damasceno@usp.br; 2 Department of Chemical Engineering, South Kensington Campus, Imperial College London, London SW7 2AZ, UK; v.hue20@imperial.ac.uk; 3 Department of Materials Physics and Mechanics, Institute of Physics, University of São Paulo, Rua do Matão 1371, São Paulo 05508-090, SP, Brazil; crmiranda@usp.br

**Keywords:** carbon nanotube, polyethylene, mechanical properties, composite

## Abstract

Advanced nanocomposite membranes incorporate nanomaterials within a polymer matrix to augment the mechanical strength of the resultant product. Characterizing these membranes through molecular modeling necessitates specialized approaches to accurately capture the length scales, time scales, and structural complexities inherent in polymers. To address these requirements, an efficient simulation protocol is proposed, utilizing coarse-grained (CG) molecular dynamics simulations to examine the mechanical properties of polyethylene/single-walled carbon nanotube (PE/SWCNT) composites. This methodology integrates CG potentials derived from the statistical associating fluid theory (SAFT-γ Mie) equation of state and a modified Tersoff potential as a model for SWCNTs. A qualitative correspondence with benchmark classical all-atom models, as well as available experimental data, is observed, alongside enhanced computational efficiency. Employing this CG model, the focus is directed at exploring the mechanical properties of PE/SWCNT composites under both tensile and compressive loading conditions. The investigation covered the influence of SWCNT size, dispersion, and weight fraction. The findings indicate that although SWCNTs enhance the mechanical strength of PE, the extent of enhancement marginally depends on the dispersion, filler size, and weight fraction. Fracture strengths may be elevated by 20% with a minor incorporation of SWCNTs. Under compression, the incorporation of SWCNTs into the composites results in a transformation from brittle to tough materials. These insights contribute to the optimization of PE/SWCNT composites, emphasizing the importance of considering multiple factors to fine-tune the desired mechanical performance.

## 1. Introduction

Polymer nanocomposite membranes have emerged as promising materials for gas separation [1], drug delivery [2], and other applications [3]. These membranes typically consist of a polymer matrix reinforced with nanomaterial fillers, where the primary role of the fillers is to enhance membrane properties, such as selectivity/permeability and mechanical strength. Among the commonly used additives, graphene and carbon nanotubes (CNTs) have been widely employed to design carbon-based membranes [4,5] owing to their exceptional tensile strength, extreme aspect ratio, and unique physical properties [6,7,8]. In general, composites exhibit desirable properties for high-performance membranes, combining the most attractive characteristics of metallic, polymeric, and ceramic systems with the outstanding properties of the fillers [8,9].

Polyethylene (PE) is a polymer of great technological interest, widely recognized for its versatility, low cost, and lightweight nature, making it suitable for a diverse range of applications in engineering and industry [10,11]. Its mechanical properties are strongly influenced by its degree of crystallinity, which varies among forms such as low-density polyethylene, high-density polyethylene, and ultra-high-molecular-weight polyethylene [11]. In recent years, significant advancements have been made to enhance the mechanical performance of various types of PE by incorporating advanced fillers.

For instance, the incorporation of CNTs into PE has been the focus of numerous research efforts, with studies demonstrating their effectiveness in improving the polymer’s mechanical properties. Zhang et al. [12] developed a novel method to disperse CNTs within a matrix of high-density polyethylene (HDPE), achieving enhanced uniformity in the composite. Salah et al. [13] investigated CNTs grown on oil fly ash as a cost-effective reinforcement material for HDPE, demonstrating the potential for large-scale, economical production of polymer nanocomposites. Additionally, Tebeta et al. [14] studied the effects of compression load on the elastic properties of HDPE/CNT composites, reporting substantial mechanical enhancements, including notable increases in tensile strength and elastic modulus, with improvements exceeding 30% for low-weight fractions of CNTs. For a comprehensive review on the mechanics of polyethylene composites, the reader is directed to [10,15,16].

Several factors influence the strength of the interfacial interaction between fillers and polymers, including the chemical composition of both structures, the size and density of the fillers, and the processing conditions during synthesis. Given the vast range of polymer and filler combinations and the numerous properties that can be tailored, computational simulations provide an efficient and cost-effective approach to systematically explore these interactions. Atomistic molecular simulations, particularly those based on molecular dynamics (MD) protocols, offer valuable insights into the behavior of polymers and composites under various thermodynamic conditions. For instance, MD simulations have been employed to study the deformation of amorphous polyethylene (PE) under tensile, compressive, and cyclic loading conditions [17,18,19]. Other studies have investigated the influence of simulation parameters, including temperature, pressure, and atomic motion, on the mechanical properties of PE [20,21,22]. Additionally, MD has been widely used to examine the mechanical properties of PE/CNT composites, providing insights into their enhanced performance through reinforcement mechanisms [23,24,25,26].

However, these detailed molecular simulations encounter challenges when attempting to model the typical characteristic length and time scales and complexity found in polymers. In this context, Coarse-Grained (CG) models present a promising avenue for investigating the properties of complex soft matter, reducing the inherent computational load by integrating out some of the detail deemed unnecessary in the study of macroscopic properties. This approach simplifies complex structures by combining a few atoms or simple molecules into segments of varying chemical groups, known as beads. Using suitable CG models, molecular simulations can handle the complexity exhibited by nanocomposite materials, which may involve phenomena occurring across large size and time scales inaccessible by all-atom models [27,28].

Building on the advantages of CG models, this study employs a CG approach based on the SAFT-γ Mie group contribution framework [29]. This framework offers a systematic and transferable method for representing molecular fluids by parametrizing interactions using experimental thermophysical properties. The SAFT-γ Mie framework has been extensively developed and applied in a series of works [30,31,32,33,34], demonstrating its versatility in accurately capturing the behavior of complex systems, including polymer systems, gas separations, and refrigerants [35]. The robustness of this methodology is further highlighted by the availability of the “Bottled SAFT” platform, a web application that provides SAFT-γ Mie force field parameters for thousands of molecular fluids [36]. This platform enhances accessibility and efficiency for researchers across various domains.

This study investigates the mechanical behavior of PE/single-walled carbon nanotube (SWCNT) composites using a hybrid CG molecular simulation protocol. For the CG model of PE, we adopted the recent parametrization proposed by Fayaz-Torshizi and Muller [37], which is based on the SAFT-γ Mie group contribution framework. This approach parametrizes interactions using experimental thermophysical data from smaller oligomers, ensuring transferability to larger polymer systems. For SWCNTs, a modified Tersoff potential was employed, with adjustments made to mitigate nonphysical failure mechanisms and accurately represent the behavior of carbon nanotube interactions.

The uniqueness of the proposed simulation protocol in the field of nanomechanics lies in employing a CG model based on the well-established SAFT-γ Mie framework. The versatility of this approach enables its extension to a wide range of polymers. Furthermore, the CG model for the nanotube has been validated against both literature data and atomistic simulations, providing a robust and reliable model that can be applied to other systems. Additionally, the investigation covered the influence of SWCNT size, dispersion, and weight fraction, offering valuable insights into their role in the mechanical behavior of polymer nanocomposites.

## 2. Methodology and Models

### 2.1. Force Fields

Force fields (FFs) are essential in molecular simulations to accurately describe interactions between atoms and molecules, playing a key role in determining material properties and behavior. Selecting the most appropriate FFs is crucial and depends on the nature of atomic interactions within the system [38].

For the atomistic model of SWCNTs, two FFs were employed: the adaptive intermolecular reactive bond order (AIREBO) [39] and the Tersoff potential [40], while the Optimized Potentials for Liquid Simulations All-Atom (OPLS-AA) force field [41] was selected for atomistic PE. The AIREBO potential has been widely used to model carbon-based nanostructures and predict their mechanical properties [42,43]. Similarly, the Tersoff potential has proven effective in modeling carbonaceous structures [44]. These two FFs were selected for comparison purposes. The OPLS-AA FF, developed by William L. Jorgensen, is designed to simulate a wide range of organic and inorganic molecules and polymers [41]. It combines quantum mechanical calculations with experimental data and is renowned for its accuracy in predicting structural and thermodynamic properties across various phases.

For the CG model of PE, we utilized the recent parametrization proposed by Fayaz-Torshizi and Muller [37]. In their study, nonbonded intermolecular parameters were derived using the SAFT-γ Mie group contribution method by minimizing an objective function that compares experimental data with the corresponding SAFT-γ Mie equation of state calculations for selected properties. SAFT-γ Mie represents an advanced version of SAFT [37], employing the Mie potential, an extended form of the Lennard-Jones potential, to describe interactions between monomers. This approach enables the construction of chain fluids with diverse monomer types and predicts their properties across a wide range of sizes and shapes. Intramolecular interactions are described using harmonic potentials. Bond stretching and angle bending parameters were determined through fully atomistic simulations using the OPLS-AA FF [37].

For the CG model of SWCNTs, we applied the FF proposed by Shang et al. [44], which is based on the Tersoff potential. This FF has demonstrated high accuracy in predicting critical properties of carbon-based structures. Further details on modifications to this potential, as well as parameter values and discussions, can be found in their study on the mechanical properties of graphene and its assemblies. In our study, we adjusted the cutoff values to mitigate nonphysical failure mechanisms commonly observed in the fracture of carbon structures, as described in [43]. The cutoff parameters employed were 4.0 Å for the upper limit and 0.0 Å for the lower limit. Finally, the cross-terms for unlike interactions among the CG beads were determined using arithmetic combining rules, ensuring consistency and compatibility within the CG model.

### 2.2. Atomistic Models

For the pristine polymer system, 125 chains of PE with 100 monomers per chain and two carbon atoms per monomer are created using Amorphous builder in MedeA [45], resulting in a system with an initial dimension of 60 Å × 60 Å × 200 Å (length × width × height) and the density of $0.809\;{g/\mathrm{cm}}^{3}$. For the case of PE/SWCNT, a long SWCNT with a tube length identical to the system height is inserted into the middle of the system, effectively crossing the boundary condition in the z-direction. To account for a density similar to the pristine PE system, the number of PE chains is reduced accordingly. The CNT has a diameter of 13.5 Å with armchair chirality and a bond length of 1.42 Å between the consecutive carbon atoms.

### 2.3. CG Model of PE and SWCNT

**Figure 1:**
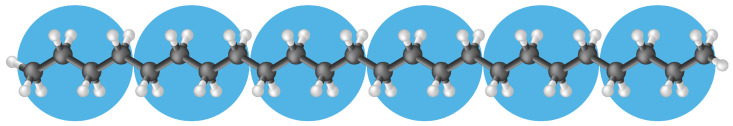
CG representation of a PE chain. Four backbone carbon atoms correspond to one CG bead (4:1 mapping scheme). The terminal groups correspond to the (CH_3_–CH_2_–CH_2_–CH_2_–) and the middle group to the (–CH_2_–CH_2_–CH_2_–CH_2_–).

The CG model of PE employs a 4:1 level of CG resolution that maps four consecutive backbone carbon atoms and the corresponding hydrogen atoms into a single bead [37]. As illustrated in Figure 1, a PE oligomer, C_24_H_50_, containing 12 monomers (74 atoms), is represented by 6 tangentially bonded beads, with each bead representing two monomers.

**Figure 2:**
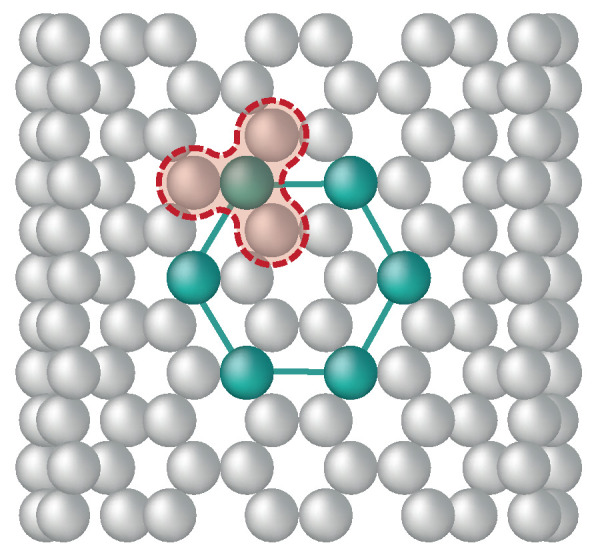
CG representation of a carbon nanotube. The green hexagonal ring illustrates the CG beads over the atomistic structure. Each bead incorporates four carbon atoms, as shown by the red highlight.

**Figure 3:**
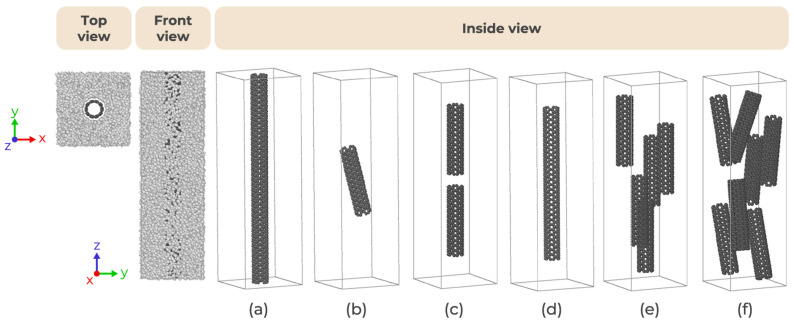
CG representations of PE/SWCNT composites with wt.% of SWCNTs of: (**a**) 11.4%, (**b**) 3.8%, (**c**) 7.4%, (**d**) 8.3%, (**e**) 17.9%, and (**f**) 25.1%. Inside view does not show the polymer molecules.

For the CG version of SWCNT, the model proposed by Ruiz et al. [46] is considered, which was originally developed to study the mechanical properties of graphene. Figure 2 shows in gray the atomistic carbon nanotube structure and in green the CG model of a hexagonal ring. Each bead represents four carbon atoms, as illustrated by the dashed red line. Notably, the CG model maintains the hexagonal symmetry characteristic of the atomistic structure, which is crucial for capturing chirality effects. This model has the same diameter and chirality as the atomistic version but with a bond length of 2.8 Å between the consecutive CG beads [44]. To match the morphology of the benchmark atomistic model, the CG PE/SWCNT system has SWCNT with identical tube length of 200 Å and PE chains with 100 monomers, 50 beads per chain, as represented by the top, front, and inside views in Figure 3a.

### 2.4. CG Model of PE/SWCNTs

Distinct concentrations of SWCNT fillers have been modeled to investigate the effects of weight percentage (wt.%) and the length of CNT fillers on the mechanical properties of the PE/SWCNT systems under tensile/compressive loading. As illustrated in Figure 3a, a long CNT is considered. Additionally, a shorter version of CNT with a tube length of 150 Å is also examined, as depicted in Figure 3d. Furthermore, one, two, five, and seven CNTs with a tube length of 70 Å have been randomly inserted into the CG PE system, as shown in Figure 3b, c, e, and f, respectively. In all cases, the number of PE chains is adjusted accordingly to maintain a similar density of PE in the systems. Noticeably, only in (a) does the CNT span the whole length of the simulation cell, being infinite in length as a virtue of the periodic boundary conditions.

### 2.5. Molecular Simulation

The polymer and SWCNT structures are modeled using the Material Exploration and Design Analysis (MedeA) simulation suite [45] and visualized using Ovito [47]. MD simulations are conducted using the LAMMPS (Large-scale Atomic/Molecular Massively Parallel Simulator) package [48] to investigate the mechanical properties of PE/SWCNT systems under tensile and compressive loading. Initially, the system energy is minimized via the conjugate-gradient method to avoid overlaps as the polymer undergoes a microcanonical ensemble (NVE) simulation at 500 K for 300 ps followed by relaxation for 50 ps using the NPT ensemble at 500 K [17]. The next relaxation cools the polymer to 300 K over a period of 50 ps with a cooling rate of 4 K/ps, followed by two additional relaxations of 50 ps at a temperature of 300 K. Periodic boundary conditions are imposed in all directions. These initial steps aim to prepare the system for the production stage, where uniaxial tensile or compressive tests are conducted by applying strain at a rate of 0.001 ps^−1^ along the length of the system (z-direction). A time step of 0.001 ps is used in the NPT ensemble for this stage. The stress values are obtained by averaging the virial stress of each atom (or CG bead), expressed by Equation (1) [49].

(1) $$\sigma_{ab}=\frac{1}{V}\sum_{\alpha }\left\{\frac{1}{2}\sum_{\beta =1}^{N}\left(R_{a}^{\beta }-R_{a}^{\alpha }\right)F_{b}^{\alpha \beta }-m^{\alpha }v_{a}^{\alpha }v_{b}^{\alpha }\right\}$$

where *a* and *b* are the Cartesian coordinate axes (x, y, or z), *N* is the number of atoms, *V* is the volume, $R_{a}^{\beta }$ indicates the location of atom β along the *a*-axis, $R_{a}^{\alpha }$ represents the location of atom α along the *a*-axis, $F_{b}^{\alpha \beta }$ denotes the force acting on atom α due to its interaction with the neighboring atom β in the *b*-direction, $m^{\alpha }$ represents the mass of atom α, and $v_{x}^{\alpha }$ and $v_{y}^{\alpha }$ are the velocities of atom α along the a- and b-directions, respectively. The strain measure is defined by

(2) $$\epsilon =\frac{\Delta L}{L^{0}}$$

where ΔL is the displacement, calculated as the difference between the current length *L* and the original length $L^{0}$.

## 3. Results

The following sections are dedicated to validating the CG SWCNTs and presenting the mechanical behavior of the PE/SWCNTs composites, examining the mechanical response of the different composites depicted in Figure 3.

### 3.1. Validation of CG Model of SWCNTs

**Figure 4:**
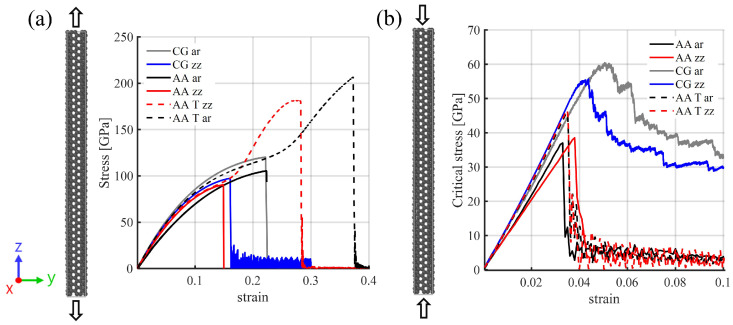
Stress–strain curves obtained for CG and atomistic (AIREBO-AA and Tersoff AA T) *ar* and *zz* nanotubes under (**a**) tensile and (**b**) compressive loading. CG models are represented by the gray and blue curves, while AA models are represented by the black and red curves and AA T by dashed lines. The black arrows represent the load direction.

Figure 4a shows the comparison of the stress–strain curves for both atomistic and CG models of armchair (*ar*) and zigzag (*zz*) SWCNTs under uniaxial tensile loading. Benchmark atomistic models are presented for both AIREBO (AA) and Tersoff potentials (AA T). The discrepancies between these two atomistic models, particularly under strain conditions, highlight the difficulties associated with the selection of forcefields for structured materials [38]. It is observed that the failure strains are similar for CG *ar* and AA *ar* models, while the fracture strength is higher for both directions compared to AA results. The overestimation observed in the stress–strain values has previously been reported in atomistic simulations of carbon nanotubes using the Tersoff potential [43,50] and is also reproduced in the same figure. Despite these differences, the proposed CG models provide appropriate predictions of the mechanical response of carbon nanotubes with an accuracy comparable to atomistic simulations. Moreover, the CG models effectively capture the effects of chirality, a crucial characteristic influencing the mechanical response of carbon nanotubes [51]. The tensile strength and failure strains for the CG SWCNTs are as follows: for *ar*, 120.2 GPa and 22%, respectively, and for *zz*, 97.4 GPa and 16%, respectively. These values fall within the range reported in the existing literature. Experimentally, ultralong carbon nanotubes have exhibited fracture strains and tensile strengths up to 17% and 200 GPa, respectively [7]. Numerically, atomistic *ar* SWCNTs typically display tensile strengths ranging from 119 to 125 GPa, while *zz* SWCNTs exhibit strengths in the range of 94–110 GPa, with fracture strains of *ar* SWCNTs falling within 18–29.6% and *zz* SWCNTs within 14–20% [52,53,54,55].

Figure 4b illustrates critical stress–strain curves for the CG and atomistic models of *ar* and *zz* SWCNTs under axial compressive loading. The *zz* and *ar* SWCNTs exhibit slightly similar values of critical stress, in good agreement with the atomistic results. However, the proposed CG model overestimates the critical stress values, as mentioned previously. The critical stress and strain values for the *ar* SWCNT are 59 GPa and 0.05, respectively, while for the *zz* SWCNT, they are 55 GPa and 0.04. These critical strain values align with literature reports for similar diameters of approximately 4% [56,57], while the critical stress for the *ar* SWCNT, considering similar diameters and force field, is approximately 57 GPa [58]. Overall, the results underscore the ability of CG models to effectively capture the mechanical response of SWCNTs despite their reduced geometrical and computational complexity.

### 3.2. Mechanical Response of PE/SWCNT Composites Under Tensile Loading

**Figure 5:**
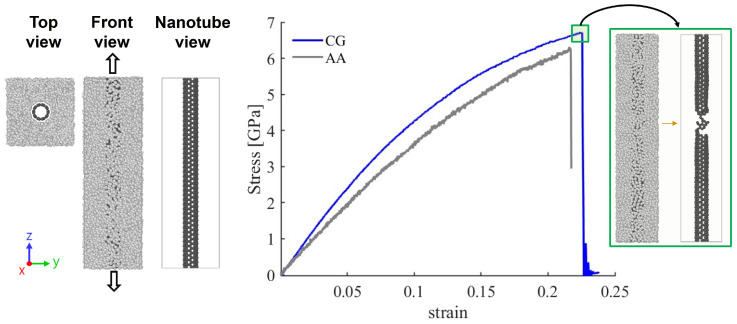
Mechanical response of PE/SWCNTs under tensile loading. On the left, the top and front views of the system are presented with the nanotube positioned inside the matrix. In the middle, a comparison between the stress–strain curves of CG and AA models is presented. The insert at the top of the CG curve highlights the mechanical response of the system at the stress–strain level, as shown on the right.

Figure 5 illustrates the mechanical response of the CG PE/SWCNT model depicted in Figure 3a. A comparison of the stress–strain curves is presented for both the atomistic (based on the AIREBO force field) and the CG models under tensile loading. The CG curve shows good agreement with the atomistic results, exhibiting slightly higher fracture strength and failure strain for strains larger than 0.04. Initially, both atomistic and CG models demonstrate linear elastic behavior, as evidenced by the linear increase in stress with strain. In the zoomed-in region on the right side of the figure, the failure region of the system is depicted, showing structural breakage in the nanotube, indicated by the abrupt decline in the stress–strain curve during simulation. It is observed that for a long continuous nanotube, with a similar polymer matrix length, the nanotube primarily bears the applied loading. The stress value of approximately 6.5 GPa falls within the range reported in the literature, which typically ranges from 6 to 11 GPa [23,59,60,61,62]. Variations in reported values in the literature can be attributed to differences in the nanotube diameter, simulation temperature, and various force fields. It is important to note that while the nanotube primarily bears the applied loading, the stress value is lower compared to an isolated nanotube, as it is calculated in relation to the matrix area, not solely the nanotube area.

In terms of computational performance, the CG model runs more than 10 times faster than the AA model. Additional details are provided in the Supplementary Materials.

### 3.3. Effect of Composite Inclusions

**Figure 6:**
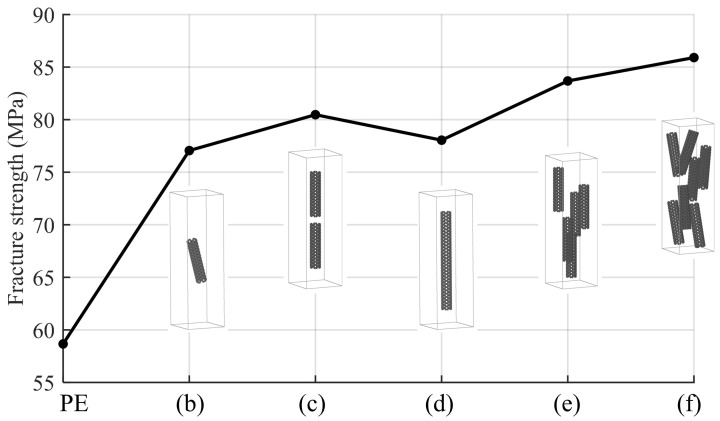
Fracture strength of CG PE/SWCNTs composites under uniaxial tensile loading. The indices (b), (c), (d), (e), and (f) on the x-axes represent the different systems described in Figure 3. In these illustrations, the polymer is removed to facilitate visualization of the nanotubes.

The previous simulations considered the singular example of a nanotube that spans the simulation box. Given the difference between the rigidity of the nanotube and the surrounding matrix, it is expected that the nanotube would be the single element that sustains the larger proportion of the strain (or compression). In a realistic composite membrane, one would expect to have inclusions of a characteristic length which are significantly shorter and/or not aligned with the direction of the extension/compression. In that sense, in this section, we explore more realistic systems, as depicted in Figure 3b–f. Here, various sizes of nanotubes are considered, randomly dispersed in the polymer matrix. Figure 6 illustrates the fracture strength for these PE/SWCNTs composites.

In comparison to PE, PE/SWCNT composites exhibit higher fracture strength, suggesting that carbon nanotubes enhance the mechanical performance of PE. However, a slight increase in fracture strength is observed with increasing SWCNT content. The graph shows an initial increase in fracture strength with the introduction of a small amount (3.8 wt%) of CNTs. This trend continues with a more substantial improvement observed at 7.4 wt%. However, at 8.3 wt%, a slight dip in fracture strength occurs, followed by a recovery and subsequent increases at 17.9 wt% and 25.1 wt%, with small differences between the latter. These findings suggest that multiple fillers increase the contact area with the polymer. However, as the number of fillers increases, the contact area between the polymer and filler decreases, leading to values similar to those with fewer CNTs wt%. Our results qualitatively agree with experimental observations on the improvement of tensile strength for low-weight fractions of carbon nanotubes [12,13] to the order of 20%. Possible discrepancies between computational simulations and experimental observations have been addressed elsewhere [23].

### 3.4. Mechanical Response of PE/SWCNTs Under Compressive Loading

**Figure 7:**
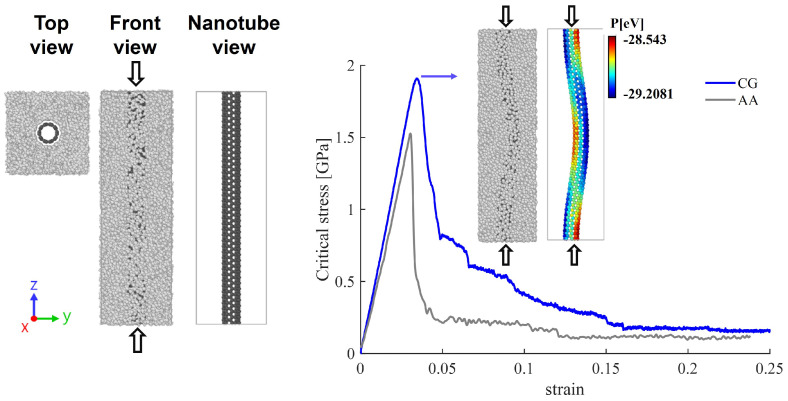
Mechanical response of AA and CG PE/SWCNT under compressive loading. On the left side, top and front views of the nanocomposite structure are presented, along with a side view illustrating the direction of the compressive force (indicated by the black arrows). The inset on the right side shows the potential energy variations of the nanotube during compression.

Figure 7 depicts the mechanical response of PE/SWCNTs under compressive loading. In this model, the nanotube spans the whole length of the polymer matrix. A comparison of the critical stress–strain curves is presented for both the AA and the CG models. The CG curve shows good agreement with the atomistic results, exhibiting slightly higher fracture strength and failure strain of 1.9 GPa and 0.034, while the AA model shows 1.5 GPa and 0.031, respectively. It is observed that the CG critical stress increases gradually with the applied load up to a strain of 0.034. Beyond this point, the curve exhibits a sharp decrease in stress, indicating material failure. The color-coded potential energy distribution in the inset view provides insight into the mechanical behavior of the nanotube. Atoms at different energy variation are depicted in a range from red (high potential energy) to blue (low potential energy), highlighting the regions where structural changes are most likely occurring as the system is compressed.

**Figure 8:**
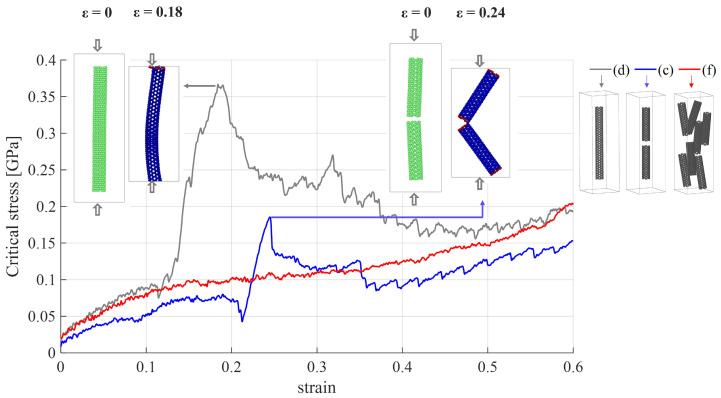
Critical stress–strain of CG models of PE/SWCNTs under compression loading. The gray curve (d) corresponds to the system shown in Figure 3d, the blue curve (c) to Figure 3c, and the red curve (f) to Figure 3f. The insets on the right show the systems. For the gray (d) and blue (c) curves, the fracture patterns of the models are presented for different strain levels.

Figure 8 presents a comparison of the critical stress–strain curves for cases (c), (d), and (f) (Figure 3). For strains up to 0.1, the critical stress of the PE/SWCNTs increases as the weight percentage of SWCNTs increases, but remains similar for systems with higher weight fractions. The behavior of the curves suggests that the nanocomposites are tough materials, exhibiting a yield point for cases (c) and (d), and no clear yield point for case (f). A more detailed explanation of these differences has been addressed elsewhere [14].

For larger strains, case (d) exhibited a significant increase in critical stress, reaching 365 MPa at a strain of 0.18. To illustrate this behavior, on the left side, Figure 8 depicts the system at ϵ=0 and ϵ=0.18. The observed peak in the curve corresponds to the moment when the simulation box is compressed to the point commensurate with the length of the nanotube, resulting in higher critical stress values. Subsequently, these values decrease to levels similar to the other models. A similar peak in the stress–strain curve is evident for case (c). At a strain of 0.24, the critical stress is 185 MPa. To illustrate this behavior, on the right side, Figure 8 illustrates the system at ϵ=0 and ϵ=0.24. At ϵ=0.24, the two carbon nanotubes collide, resulting in an increase in stress. In the system with seven nanotubes (case (f)), no peak is observed in the stress–strain curve. The stress of 100 MPa is sustained until a strain of 0.3, with a slight increase for higher strain levels.

## 4. Conclusions

Our study focused on investigating the mechanical properties of polyethylene reinforced with single-walled carbon nanotubes under both tension and compression loading conditions. Through extensive molecular modeling, we examined the influence of various factors, including CNT size, dispersion, and weight fraction, on the mechanical behavior of the composites. Moreover, we provided a comparison of the mechanical properties between the coarse-grained and atomistic models. CG models offered substantial computational advantages over the atomistic system, demonstrating superior efficiency without compromising accuracy. Using the proposed CG model, we found that the presence of CNTs can enhance the mechanical strength of PE, but this enhancement has a complex relationship with dispersion, filler size, and weight fraction. These factors have a direct influence on the mechanical behavior of the composites, leading to a transition from brittle to tough materials.

These results confirm the potential of carbon nanotubes to improve the mechanical properties of PE, particularly under compression loading. Moreover, they offer valuable insights into how various factors, such as size, dispersion, and weight fraction, directly influence the material’s behavior, ultimately determining whether it exhibits characteristics of brittle or tough materials. Additionally, it is noteworthy that the stress–strain values exhibit a complex relationship with the deformation level, size of the nanotube, and dispersion. This underscores the intricate interplay of these factors in dictating the mechanical response of the system, highlighting the need for efficient modeling approaches. The developed protocol is versatile and can be adapted to study a wide range of polymer/carbon structures, providing a robust tool to explore their mechanical properties under various thermodynamic and loading conditions.

## Data Availability

Data are contained within the article and Supplementary Materials.

## Supplementary Materials

The following supporting information can be downloaded at https://doi.org/10.6084/m9.figshare.25939642. Supplementary information is provided with this manuscript, including comparative analysis of computational time and example input files.
